# Genetic integrity and individual identification-based population size estimate of the endangered long-tailed goral, *Naemorhedus caudatus* from Seoraksan National Park in South Korea, based on a non-invasive genetic approach

**DOI:** 10.1080/19768354.2020.1784273

**Published:** 2020-06-25

**Authors:** Ji Eun Jang, Nam Hyeong Kim, Sangjin Lim, Ki Yoon Kim, Hyuk Je Lee, Yung Chul Park

**Affiliations:** a Molecular Ecology and Evolution Laboratory, Department of Biological Science, College of Science and Engineering, Sangji University, Wonju, Korea; b Department of Forest Environment Protection, College of Forest and Environmental Sciences, Kangwon National University, Chuncheon, Korea

**Keywords:** Korean goral, molecular scatology, non-invasive sampling, population size estimate, probability of identity

## Abstract

The long-tailed goral (also called the Amur goral) *Naemorhedus caudatus* (subfamily Caprinae), a vulnerable and protected species designated by IUCN and CITES, has sharply been declining in the population size and is now becoming critically endangered in South Korea. This species has been conserved as a natural monument by the Korean Cultural Heritage Administration since 1968. In this study, using 78 fecal DNA samples with a non-invasive genetic approach, we assessed the genetic integrity and individual identification-based population size for the goral population from Seoraksan National Park representing the largest wild population in Korea. Using the successfully isolated 38 fecal DNA, phylogeographic and population genetic analyses were performed with mitochondrial DNA control region (*CR*) sequences and nine microsatellite loci. We found seven *CR* haplotypes, of which five were unique to the Seoraksan population, considering previously determined haplotypes in Korean populations. The Seoraksan population showed higher haplotype diversity (0.777 ± 0.062) and mean number of alleles (4.67 ± 1.563) relative to southern populations in Korea reported from previous studies, with no signal of a population bottleneck. Microsatellite-based individual identification estimate based on probability of identity (PID) indicated a population size of ≥30 in this population. Altogether, we suggest that for future management efforts of this species in the Seoraksan National Park, conserving its genetic integrity as an ‘endemic’ lineage, and curbing a decrease in its number through mitigating habitat destruction might be key to secure the population for the long term.

## Introduction

Non-invasive genetic monitoring has become popular for last few decades as it largely complements limitations of traditional monitoring techniques, such as *in situ* field survey and radio telemetry, which usually require capturing the target species (Schwartz et al. 2007; Rodgers and Janečka 2013). It is now recognized as a versatile tool for conserving various wild mammals and birds. The genetic data obtained from non-invasive samples (e.g. feces and hairs) can be applied to a diverse array of research areas. Fecal DNA analysis allows for the identification of species, individuals, and even sex, particularly for ‘hard-to-catch’ species like threatened wild mammals (Mondol et al. 2009; Moran-Luis et al. 2014). Therefore, it has been proven to be a useful approach for understanding ecological features of the study species, such as its behavioral range, distribution pattern and abundance. Non-invasive genetic method can also be used to perform dietary analysis for understanding trophic niche of predators, which is accomplished by identifying what prey items they feed on. This dietary information provides a practical support for the recovery of endangered populations (Deagle et al. 2009). Furthermore, it has been used for phylogeographic (Mukherjee et al. 2010), and population and landscape genetic (Sharma et al. 2011; Choi et al. 2015) studies of wild animals (Walker 2005). Hence, non-invasive genetic approach to species that are difficult or sometimes impossible to be monitored in the wild is essential for conservation efforts for endangered populations. It is valuable, particularly when other non-invasive techniques, such as camera traps, track plate and scent stations are inaccessible.

**Figure 1.**
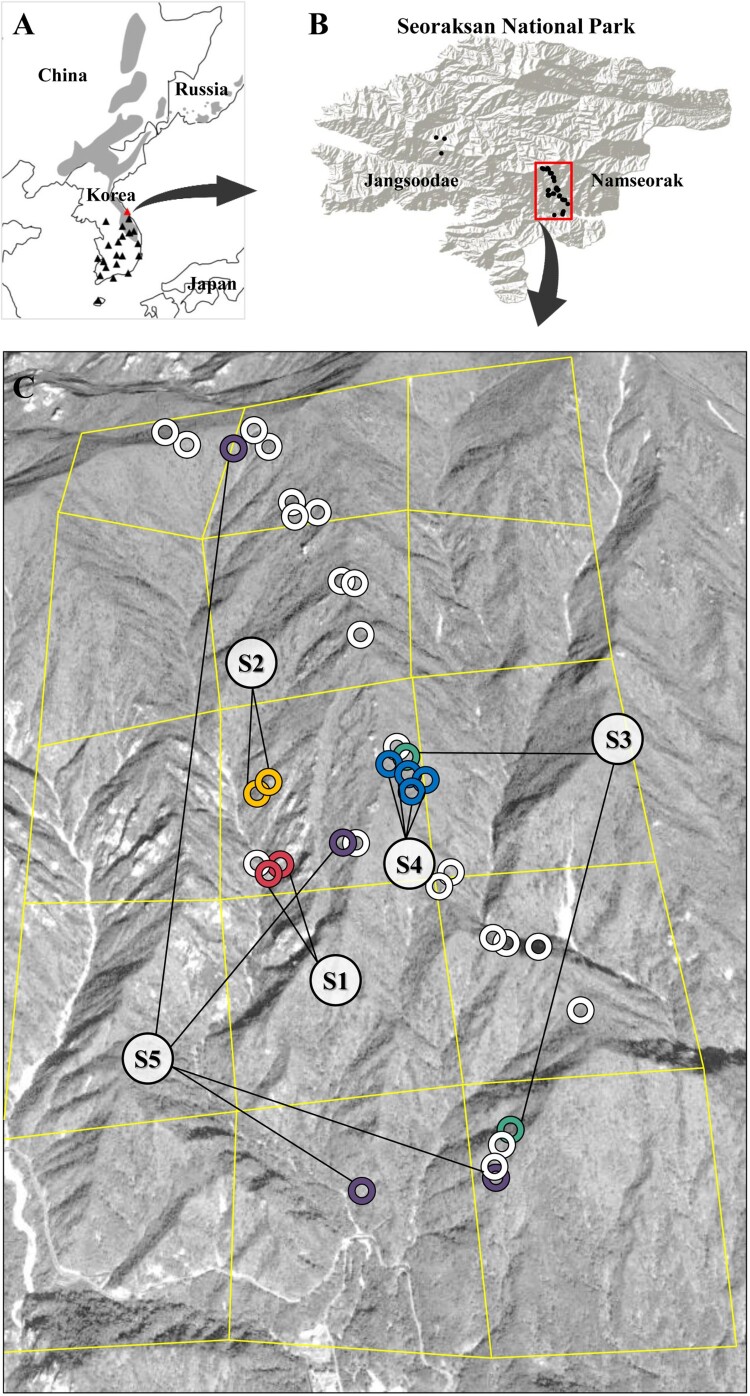
Sampling localities of *N. caudatus* for non-invasive fecal DNA samples. (A) The geographic distribution of *N. caudatus* (in gray) was modified from a map in Duckworth et al. (2008). Triangles in black represent national parks located in South Korea. A triangle in red represents Seoraksan National Park where fecal samples were collected in this study. (B) Two sampling localities [Jangsoodae (*N* = 3) and Namseorak (*N* = 35)] within Seoraksan National Park. (C) Sampling spots for 30 individuals (38 fecal samples) from Namseorak. Small circles in different colors (in red, yellow, green, blue and purple) indicate different individuals (genotypes) except that each of those in white represents genetically different individuals. Squares with yellow grids are approximately 800 m^2^.

The long-tailed goral (also known as the Amur goral), *Naemorhedus caudatus* (subfamily Caprinae) is a protected species classified as vulnerable on IUCN (International Union for Conservation of Nature) (Duckworth et al. 2008) as well as listed on Appendix I of CITES (Convention on International Trade in Endangered Species of Wild Fauna and Flora), due to its sharp declining in the population size primarily by habitat destruction and fragmentation. This species is distributed partly in the southeastern Russia, northeastern China and also the Korean Peninsula (Grubb 2005) (Figure 1(A)). In South Korea, the long-tailed goral has been categorized as an endangered wild species class I by the South Korean Ministry of Environment, and a natural monument (No. 217) by the Korean Cultural Heritage Administration for the legal protection of this species. Nevertheless, Korean goral populations have been continually decreasing most likely due to increasing anthropogenic pressure, such as poaching, habitat devastation and fragmentation (Cultural Heritage Administration 2011).

South Korean *N. caudatus* populations are expected to be under a serious threat of extermination within the next 20 years due to genetic problems, such as inbreeding and genetic drift with a small number of effective population sizes (*N*_e_) (Yang 2002). In a previous study of goral populations covering a wide range of South Korea, a total of 690–784 individuals were suggested to be present (Yang 2002). Only a few habitats [e.g. Seoraksan National Park, demilitarized zone (DMZ), Yanggu-Hwacheon and Uljin-Samcheok-Bonghwa regions] are considered as housing stable goral populations (where more than 100 individuals would persist), while the remaining populations are suggested to be comprised of less than 10 individuals. Amongst these regions, the Seoraksan National Park is the most widely ranged habitat for the goral, as at least 155 individuals were corroborated to occur within this area (Species Restoration Technology Institute 2009). This national park is also well known as a famous attraction place, where the largest number of tourists visit annually (about five million visitors per year) and high anthropogenic pressure would thus be expected. Hence, there is an urgent need for conservation and management of this endangered species in this area.

While several studies have been conducted for wild mammals (e.g. leopard cat, otter) and birds using a non-invasive genetic approach to individual identification (Park et al. 2011; Joo and Park 2012; Lee et al. 2013), this method has not yet been applied to wild Korean goral. As an effort to protect the Korean goral, population and conservation genetic studies have been performed during recent years since early 2000s, for example, a phylogenetic reconstruction using mitochondrial DNA (mtDNA) cytochrome *b* (*cyt b*) (Min et al. 2004), the development of novel microsatellite markers (An et al. 2005; An, Choi, et al. 2010), mtDNA control region (*CR*)-based phylogeography (An, Okumura, et al. 2010), and analyses of genetic diversity and population structure (Choi et al. 2015). However, for estimating the population size of *N. caudatus* in South Korea, camera traps have mainly been used while non-invasive genetic approach has been overlooked. Moreover, most genetic studies using non-invasive samples from Korean goral populations used a limited number of samples collected from small number of natural populations.

Noninvasive genotyping with microsatellites typically needs multiple PCR replicates to avoid possible genotyping errors during data collection (Navidi et al. 1992; Miquel et al. 2006). However, given multiple genotyping is time-consuming and expensive processes a number of studies have recently attempted to compromise the limitations of a single tube method under several experimental issues taken into account (Rutledge et al. 2009; Ramón-Laca et al. 2015). The suggested considerations include sampling time for minimizing DNA degradation/contamination (Piggott and Taylor 2003; Piggott 2005), isolation of only endogenous DNA for avoiding the presence of PCR inhibitors, and the use of a shorter length and less number of repeat motif of microsatellite loci genotyped (Broquet et al. 2006).

Here, by utilizing non-invasive samples of *N. caudatus* we first report on individual identification-based population size estimate for its largest wild South Korean population, Seoraksan National Park. Using mtDNA *CR* and nine microsatellite loci, we further assessed the genetic integrity, level of genetic diversity and *N*_e_ for the Seoraksan population and also tested whether this population has experienced a recent genetic bottleneck. The results of our study will assist in developing conservation strategies for the effective managements of this species in the future.

## Materials and methods

### Sample collection and goral species identification

A total of 78 fecal samples were collected from two localities (Jangsoodae and Namseorak) at Seoraksan National Park in South Korea between 2015 and 2016 mainly during a winter season (Figure 1(B,C)). Every collected sample was stored at −20°C until genetic analysis. Genomic DNA was extracted from the outer parts of fecal samples using a DNeasy Blood and Tissue Kit (Qiagen) to obtain only endogenous DNA from epithelial cells of the intestine wall (Rutledge et al. 2009; Ramón-Laca et al. 2015). To test whether the obtained fecal samples belonged to gorals, polymerase chain reaction (PCR) was used to amplify the mtDNA *cyt b* with forward (ArtF: 5-CCCCATCAAAYATCTCATCAT-3) and reverse (ArtR: 5-TCGACTGGYTGKCCTCCAATTC-3) primers, which were designed for the long-tailed goral (*N. caudatus*) based on four sequences of phylogenetically related species (*Capreolus pygargus, Hydropotes inermis argyropus, Capra hircus* and *N. caudatus*) of Artiodactyla available from GenBank database (accession nos.: KJ681492, JF802125, KM670319 and MG865962). PCR cycling conditions comprised an initial denaturation phase at 94°C for 5 min, followed by 35 cycles of 94°C for 20 s, 54°C for 30 s, and 72°C for 30 s, followed by a terminal extension phase at 72°C for 12 min. Only 43 of the samples were successfully amplified for the mtDNA *cyt b* gene (472 bp). By using BLAST (basic local alignment search tool), we confirmed that the obtained *cyt b* sequences matched with *N. caudatus* with the highest ident value (99.9%).

## MtDNA control region (CR) sequencing and data analyses

The mtDNA *CR* region (592 bp) was amplified with the Cytb up and NH16129 primers (An, Okumura, et al. 2010) for only 43 fecal samples identified as gorals from the *cyt b* sequences. A total of 38 *CR* sequences obtained (five samples could not be amplified for *CR*) were aligned using Clustal W in BIOEDIT v7.2.5 (Hall 1999) and manually verified. A haplotype network was constructed using HAPSTAR v0.7 (Teacher and Griffiths 2011) with seven haplotypes found in this study, along with 12 haplotypes found in a previous study of long-tailed gorals in South Korea [accession nos.: EU259152-EU259176 (An, Okumura, et al. 2010); the 25 haplotypes were merged into 12 haplotypes due to adjustments to a shorter length of 592 bp]. The number of haplotypes (*N*_H_) and polymorphic sites (*N*_P_), haplotype diversity (*h*) and nucleotide diversity (*π*) were estimated using ARLEQUIN v3.5.1 (Excoffier and Lischer 2010). MtDNA sequences of the seven haplotypes found in this study can be accessed via GenBank with accession nos. MT084798-MT084804 (http://ncbi.nlm.nih.gov/nuccore).

### Microsatellite genotyping and data analyses

The nine published primer sets (SY3A, SY12A, SY12B, SY48, SY58, SY71, SY76, SY84B and SY129), which were designed for the long-tailed goral *N. caudatus* (An et al. 2005; An, Choi, et al. 2010), were used for microsatellite genotyping of our samples. We chose the primers that amplify short fragments of microsatellites (a range of 93∼288 bp) with a repeat motif of dinucleotide (except SY12B, SY71 and SY84B) because genotyping error rates tend to increase with a length and number of repeat motif (Broquet et al. 2006). PCR of 38 fecal samples was performed using three primer sets, which were labeled with fluorescent dyes (FAM, HEX and TAMRA) on forward primers, using a Multiplex PCR Kit (Qiagen), with the following conditions: an initial denaturation at 94°C for 15 min; followed by 35 cycles of 94°C for 30 s, 60°C for 1 min 30 s, and 72°C for 1 min; and a final extension step of 60°C for 30 min. Fragment sizes were determined with the ROX 500 bp size standard (ABI) using GENEMAPPER software v5.0 (Applied Biosystems).

The number of alleles across loci (*N*_A_), observed (*H*_O_) and expected (*H*_E_) heterozygosity and inbreeding coefficient (*F*_IS_) were estimated using GENEPOP v.4.0 (Rousset 2008) and FSTAT v.2.9.3.2 (Goudet 2001). The presence of null alleles was investigated using MICROCHECKER v2.2.3 (Van Oosterhout et al. 2004) with 1000 randomizations at the 95% confidence level. *P*-value of multi-locus tests for Hardy-Weinberg equilibrium (HWE) and linkage disequilibrium (LD) tests for genotypes among pairs of nine loci were calculated in GENEPOP using a Bonferroni correction.

A Wilcoxon sign-rank test of genetic bottleneck or founder effect was then performed using the two-phase mutation (TPM) model in BOTTLENECK v1.2.02 (Piry et al. 1999) by the occurrence of a mode shift and/or significant excess of heterozygosity. Contemporary *N*_e_ were also calculated for the Seoraksan population based on the LD method implemented in NEESTIMATOR v2.01 (Do et al. 2014). To examine a resolving power for individual identification that different genetic individuals are identified as the same individuals, we calculated probability of identity (P_ID_; probability that two non-related individuals share the same genotypes) and P_ID_ for siblings (P_IDsib_; probability that siblings share the same genotypes) in GIMLET v1.3.3 (Valière 2002).

## Results

### Phylogeographic relationships of Korean goral populations

**Figure 2.**
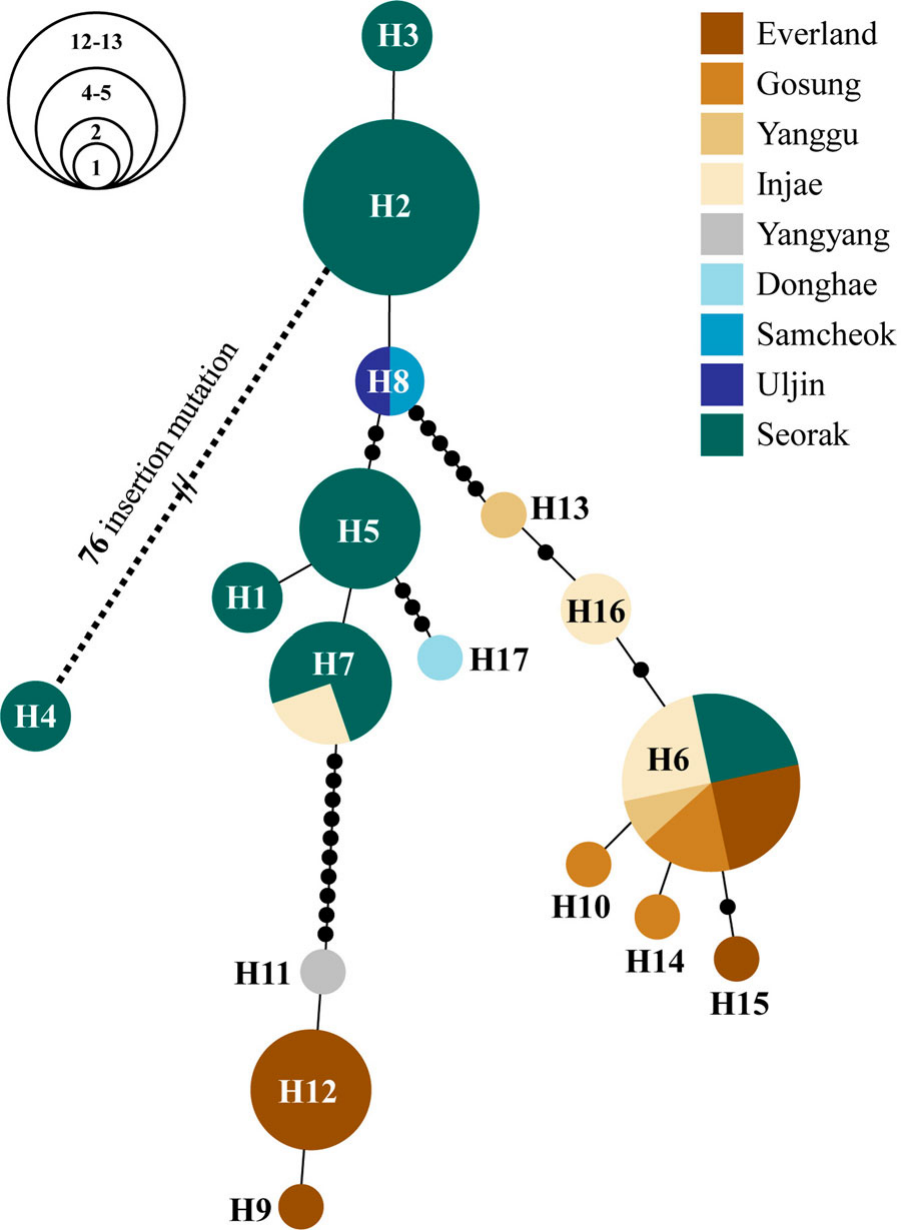
Haplotype network of seven *CR* haplotypes (592 bp) detected in this study along with 12 haplotypes determined in a previous study (An, Okumura, et al. 2010) of *N. caudatus* from South Korea, including eight localities (Everland, Gosung, Yanggu, Injae, Yangyang, Donghae, Samcheok and Uljin). Different colors represent different localities. Each line in the network represents a single mutation step between haplotypes, irrespective of its length. Small circles in black denote intermediate haplotypes that are not present in our samples, but are necessary to connect the observed haplotypes to the network. The circle size is proportional to individual numbers that belong to the respective haplotypes.

To investigate phylogeographic relationships of the Korean goral populations, we used previously determined 12 haplotypes (An, Okumura, et al. 2010) of *N. caudatus* from eight localities in South Korea, including Everland, Gosung, Yanggu, Injae, Yangyang, Donghae, Samcheok and Uljin as well as seven haplotypes (H1-H7) detected in this study. Only two haplotypes (H6, H7) found in this study were identical to the previously determined haplotypes, while the remaining five haplotypes have never been detected before, suggesting they are unique to the Seoraksan National Park region (Figure 2). Haplotype network of 17 haplotypes from nine Korean goral populations was linked by 1–19 mutational steps if the 76 insertions in H4 (in reference to H2) were considered a single mutational step.

### Genetic diversity

For the *CR* gene fragments of 38 fecal samples representing 30 genetic individuals (see below) of *N. caudatus* from Seoraksan National Park, seven haplotypes were detected with 89 polymorphic sites including 76 indel mutation (Table 1). Estimates of *h* and π were 0.777 ± 0.062 and 0.022 ± 0.012, respectively. Among the seven haplotypes, the most common haplotype (H2) was shared by 13 individuals (43.3%), and the next most frequent haplotype (H5) had 16.7% of the total sampled individuals. The five haplotypes (H1-H5) were found to be unique haplotypes (i.e. private haplotypes) that can be only observed in Seoraksan National Park.

**Table 1.** Summary of the level of genetic diversity in *N. caudatus* from Seoraksan National Park using 30 individuals estimated from 38 fecal samples at mtDNA *CR* sequences and nine microsatellite loci.

mtDNA *CR* (592 bp)				Nine microsatellites				
*N*_H_	*N*_P_	*h*	*π*	*N*_A_	*H*_E_	*H*_O_	*F*_IS_	HWE
7	89	0.777 ± 0.062	0.022 ± 0.012	4.67	0.559 ± 0.127	0.502 ± 0.106	0.103 ± 0.145	0.011

Note: *N*_H_: number of haplotypes; *N*_P_: number of polymorphic sites; *h*: haplotype diversity; *π*: nucleotide diversity; *N*_A_: observed mean number of alleles across nine loci; *H*_E_: expected heterozygosity; *H*_O_: observed heterozygosity; *F*_IS_: inbreeding coefficient; HWE: *P* values for multilocus tests for Hardy-Weinberg Equilibrium.

For nine microsatellite loci, *N*_A_ across the loci was 4.67 ± 1.563, and *H*_E_ and *H*_O_were 0.559 ± 0.127 and 0.502 ± 0.106, respectively (Table 1). The *F*_IS_ value was 0.103, indicating the possibility of inbreeding within the Seoraksan population of *N. caudatus*. The estimated frequencies of null alleles across the loci (except SY12B) were 0.199, ranging from 0.000 (SY58) to 0.439 (SY84B), suggesting very low to moderate levels of probability of null alleles. Based on multi-locus tests for HWE expectations, the possibility of non-random mating (e.g. inbreeding) could not be rejected (*P* = 0.011). Tests of genotypic LD showed no significant allelic association between the nine loci (*P* > 0.05) [except SY76 versus SY3A and SY12A (*P* < 0.05)] after the Bonferroni correction.

### Population bottleneck test

A BOTTLENECK analysis of nine microsatellite genotypes showed that the expected number of loci were five for a deficiency of heterozygosity and four for an excess. Allelic frequency distribution showed a normal L-shaped distribution with a probability of 0.320. Therefore, null hypothesis could not be rejected with a Wilcoxon sign rank test, suggesting the study population is unlikely to have experienced a recent genetic bottleneck.

### Individual identification-based population size estimate and *N_e_*

The nine loci genotyped for 38 non-invasive samples showed the extremely low levels of probability of identity (P_ID_: 2.0 × 10^−4^; P_IDsib_: 2.4 × 10^−2^) (Table 2), suggesting two randomly chosen individuals are highly likely to have different genotypes at the loci tested (Waits et al. 2001). Seven of the 9 loci showed no allelic dropout, while the remaining two loci revealed dropout rates of 10.3–13.8% from seven samples (Table 2).

**Table 2.** Genetic variability at nine microsatellites for 38 fecal samples of *N. caudatus* from Seoraksan National Park.

Locus	*N*_AP_	*H*_E_	*H*_O_	P_ID_	P_IDsib_
SY3A	4.93	0.640	0.500	0.001	0.043
SY58	4.00	0.369	0.357	0.000	0.004
SY12B	3.00	0.392	0.467	0.000	0.009
SY48	2.00	0.506	0.517	0.000	0.014
SY84B	4.97	0.676	0.552	0.019	0.188
SY71	5.00	0.462	0.378	0.000	0.006
SY129	4.93	0.560	0.448	0.000	0.024
SY76	7.93	0.736	0.567	0.115	0.414
SY12A	4.93	0.689	0.733	0.003	0.087
Across loci	4.67	0.559	0.502	0.000	0.024

Note: *N*_AP_: observed number of alleles per locus, *H*_E_: expected heterozygosity, *H*_O_: observed heterozygosity, P_ID_: probability of identity among multilocus genotypes (MLG), P_IDsib_: probability of identity among siblings.

Assuming that identical genotypes for all the nine loci analyzed were considered as the same individuals, we found five cases of identical nine loci genotypes among 38 fecal samples: two fecal samples (S1 in red); two (S2 in yellow); two (S3 in green); four (S4 in blue); four (S5 in purple) (Figure 1(C)). Therefore, the nine samples (each of two samples for S1, S2, and S3, and each of three samples for S4 and S5) were presumed to be from the same individuals, as the probability of sharing identical genotypes between two different individuals was very low (P_ID_ < 0.001). Unexpectedly, however, the nine loci genotypes from the fecal samples could not 100% match with the *CR* haplotypes from the same individuals. In fact, two samples of S3 had different *CR* haplotypes, H1 and H4, respectively, suggesting they represent genetically different individuals. Therefore, we finally determined at least 30 Korean goral individuals in Seoraksan National Park. *N*_e_ estimate for the Seoraksan population was only a median of 10.2 with 95% confidence interval of 6.6–16.2.

## Discussion

The sharply decreasing population of the long-tailed goral becomes a protected species worldwide and also critically endangered in South Korea. Here, we show that the goral population from Seoraksan National Park, which is one of the largest populations in South Korea, has evolved its own genetic integrity of multiple ‘endemic’ matrilineal lineages with rather a fairly high haplotype and microsatellite diversity. Our study also finds that this particular population has at least more than 30 genetically different individuals from the areas that we sampled.

However, multiple genotyping has been suggested to obtain reliable microsatellite dataset for fecal samples (Navidi et al. 1992; Miquel et al. 2006). Limitations of noninvasive genotyping result from low DNA quality (i.e. degraded DNA), poor extract quality (i.e. the presence of PCR inhibitors), and/or a high risk of DNA contamination. Although only a single tube method is applied in this study, we considered the suggested experimental recommendations to overcome these limitations. First, a majority of fecal samples (81.6%) used for the genetic analysis were sampled during a winter season, which could help minimizing DNA degradation and contamination of fecal specimens (Piggott and Taylor 2003; Piggott 2005). Second, to exclude PCR inhibitors as much as possible we used the outer parts of fecal samples for DNA extraction to obtain only purely endogenous DNA from epithelial cells of the intestine wall (Rutledge et al. 2009; Ramón-Laca et al. 2015). Since genotyping error rates increase with a length and number of repeat motif, we used short fragments of microsatellites (a range of 93∼288 bp) with a repeat motif of dinucleotide (except SY12B, SY71 and SY84B) (Broquet et al. 2006). Our estimated null allele frequencies of 0.199 across the loci would allow for verifying the reliability of our dataset. Nevertheless, multiple-tubes approach would surely provide more reliable genotype data.

A total of seventeen mtDNA *CR* haplotypes found in this (7 haplotypes) and previous (An, Okumura, et al. 2010) studies show that the Seoraksan goral population has greater *N*_H_ than any other Korean populations studied so far. These results suggest that the Seoraksan population has a relatively large *N*_e_ compared to other Korean populations. The Seoraksan population’s having five unique haplotypes represents its own genetic integrity or evolutionary lineage. A previous study suggests that private haplotypes may sometimes represent environment specific local adaptation of populations, albeit certainly depending on loci tested (Sjöstrand et al. 2014). Our phylogeographic analysis of this population finds five new mtDNA *CR* haplotypes (H1-H5; Figure 2) that were not seen in previous analyses (An, Okumura, et al. 2010). While a recent report (Species Restoration Technology Institute 2013) that 36 individuals from six localities in South Korea share an identical single *CR* haplotype, the Seoraksan population houses a much greater level of mtDNA diversity. Hence, greater conservation efforts should be made to maintain the observed ‘endemic’ Seoraksan National Park lineages (e.g. private haplotypes). However, further investigation of phylogeographic relationships in this species, including recently published dataset containing longer sequences of 1099 bp obtained from populations of Injae, Gosung, Yanggu and Hwacheon located in the northern parts of South Korea (Chun 2015) will be required.

The level of genetic diversity in the Seoraksan population appears to be fairly high. Seven *CR* haplotypes were observed with *h *= 0.777 and *π *= 0.022 and *N*_A_ estimate based on 9 microsatellites was 4.64 (Table 1). The level of *h* found in this study shows an intermediate level between the northern (*h *= 0.926) and southern (0.609) parts of South Korea documented in a recent study that found 22 *CR* haplotypes from 66 *N. caudatus* individuals (Chun 2015). Although the level of allelic richness could not be directly compared, we also find that the level of *N*_A_ is slightly lower than those of two earlier studies [5.38 (Cultural Heritage Administration 2011) and 5.08 (Choi et al. 2015)] of 13 microsatellites for Korean goral populations from the northern part of South Korea including Seoraksan National Park. A positive value of *F*_IS _= 0.103 with a statistical significance cannot allow us to exclude the possibility of non-random mating, indicative of inbreeding. Nonetheless, no genetic signal of a population bottleneck was suggested, which is inconsistent with a previous study (Choi et al. 2015). A rapid population decline would not be expected, as this national park has been a protected area with illegal poaching prohibited since 1968. From a population genetic perspective, Seoraksan National Park region seems to have relatively well-established goral population and therefore there should be a need for the effective conservation and management of this particular population. Maintaining this population demographically stable with no human interference might be important for conserving this local population.

The observed minimum number (*N* ≥ 30) of genetically defined individual gorals in Seoraksan National Park is lower than would be expected. In a combination of feces tracking and camera traps, the population size in this region was found to range from 160 to 251 individuals (Choi et al. 2015). 8149 photographs obtained by camera traps showed a total of 88 individuals (29 from Jangsoodae and 59 from Namseorak) (Yung Chul Park, unpublished data). A discrepancy in the population size of Seoraksan National Park can be explained by the fact that our samples analyzed were collected exclusively from Namseorak region. A study showed that only scat samples gathered in winter season is feasible for DNA extraction with a high success rate of PCR amplification (Lonsinger et al. 2015). Unfortunately, only 43 out of 78 fecal samples collected in this study were well-preserved and successful for DNA extraction. Therefore, a small number of sample sizes analyzed can affect the observed discrepancy. To solve this problem, it is required to conduct further analyses on more fecal DNA samples using a recently developed method of improving the success rates of DNA extraction and genotyping (e.g. Panasci et al. 2011).

Based on the results of this study, we suggest that future conservation management efforts of endangered long-tailed goral of the Seoraksan population should involve maintaining the ‘endemic lineage (Seoraksan National Park lineage)’ to prevent the loss of its genetic integrity (Antognazza et al. 2016), and also minimizing possible damage on the current habitat environments. However, since Seoraksan National Park is just one of the local habitats for species distribution of *N. caudatus*, it is necessary to extend a research into an investigation for the entire Korean goral populations. Our study needs further investigation of multi-tube genotyping with more vigorous analyses on dataset, such as allelic dropout, false (or null) alleles, and etc. Ideally, using genome-wide scans or mtDNA and microsatellites as genetic markers, population genetic (genomic) assessments with non-invasive genetic approach using a much larger number of fecal DNA samples should be used to more accurately identify the number of individuals within populations and also determine the level of gene flow among populations in order to help to design the effective management strategies.
